# Reactive Oxygen Species Production and Mitochondrial Dysfunction Contribute to Quercetin Induced Death in *Leishmania amazonensis*

**DOI:** 10.1371/journal.pone.0014666

**Published:** 2011-02-08

**Authors:** Fernanda Fonseca-Silva, Job D. F. Inacio, Marilene M. Canto-Cavalheiro, Elmo Eduardo Almeida-Amaral

**Affiliations:** Laboratório de Bioquímica de Tripanosomatideos, Instituto Oswaldo Cruz (IOC), Fundação Oswaldo Cruz (FIOCRUZ), Rio de Janeiro, Rio de Janeiro, Brazil; New York University, United States of America

## Abstract

**Background:**

Leishmaniasis, a parasitic disease caused by protozoa of the genus *Leishmania*, affects more than 12 million people worldwide. Quercetin has generated considerable interest as a pharmaceutical compound with a wide range of therapeutic activities. One such activity is exhibited against the bloodstream parasite *Trypanosoma brucei* and amastigotes of *Leishmania donovani*. However, the mechanism of protozoan action of quercetin has not been studied.

**Methodology/Principal Findings:**

In the present study, we report here the mechanism for the antileishmanial activity of quercetin against *Leishmania amazonensis* promastigotes. Quercetin inhibited *L. amazonensis* promastigote growth in a dose- and time- dependent manner beginning at 48 hours of treatment and with maximum growth inhibition observed at 96 hours. The IC_50_ for quercetin at 48 hours was 31.4 µM. Quercetin increased ROS generation in a dose-dependent manner after 48 hours of treatment. The antioxidant GSH and NAC each significantly reduced quercetin-induced cell death. In addition, quercetin caused mitochondrial dysfunction due to collapse of mitochondrial membrane potential.

**Conclusions/Significance:**

The effects of several drugs that interfere directly with mitochondrial physiology in parasites such as *Leishmania* have been described. The unique mitochondrial features of *Leishmania* make this organelle an ideal drug target while minimizing toxicity. Quercetin has been described as a pro-oxidant, generating ROS which are responsible for cell death in some cancer cells. Mitochondrial membrane potential loss can be brought about by ROS added directly *in vitro* or induced by chemical agents. Taken together, our results demonstrate that quercetin eventually exerts its antileishmanial effect on *L. amazonensis* promastigotes due to the generation of ROS and disrupted parasite mitochondrial function.

## Introduction

Recently, the effects of several drugs that interfere directly with mitochondrial physiology in parasites such as *Leishmania* have been described [1], [2]. The unique mitochondrial features of *Leishmania* make this organelle an ideal drug target while minimizing toxicity. *Leishmania* has a single large mitochondrion which is distributed in branches under the subpelicular microtubes and a specialized region rich in DNA called the kinetoplast [3].

Leishmaniasis, a parasitic disease caused by protozoa of the genus *Leishmania*, affects more than 12 million people worldwide [4]. Treatment of leishmaniasis is based on pentavalent antimonials, drugs developed more than 50 years ago that are toxic and prone to drug resistance [5]. Several drug screens of natural compounds have been successful in discovering novel compounds for treating some parasitic diseases [6]. Extracts obtained from plants, as well as pure compounds including some kinds of flavonoids, have been reported to possess significant antiprotozoan activities [7]–[9].

Quercetin is the most common polyphenolic flavonoids present in plants such as onions, ginko biloba and tea and can be absorbed by humans. Quercetin has a wide range of reported biological effects including antioxidant, anti-hypertension, anti-inflammatory, antimicrobial and antiprotozoan activities [10], [11], although, the precise molecular mechanism of action of quercetin has not yet been demonstrated. Quercetin can induce the production of superoxide anion, hydrogen peroxide, and other reactive oxygen species (ROS) [12]–[15].

ROS are generated in cells infected by pathogens to combat infection. ROS can also be generated in response to some drugs, and the same principle works for certain antiprotozoan drugs in killing parasites in an infected cell. This property of a drug, to generate ROS to cause the destruction of cellular macromolecular components, is important because this action can be modulated to derive maximal effect.

The aim of the present work is to study the antileishmanial activity of quercetin and describe the mechanism of quercetin against promastigotes of *Leishmania amazonensis*. Our results demonstrate that the effect of the quercetin is associated with ROS production leading to mitochondrial dysfunction, ultimately causing parasite death.

## Materials and Methods

### 1. Reagents

H_2_DCFDA (2′,7′-dichlorodihydroflurescein diacetate) was obtained from Invitrogen Molecular Probes (Leiden, The Netherlands). Others reagents were purchased from Merck (São Paulo, Brazil) or Sigma-Aldrich (St Louis, MO). Deionized distilled water was obtained using a MilliQ system of resins (Millipore Corp., Bedford, MA) and was used in the preparation of all solutions.

### 2. Parasites

Promastigotes of *L. amazonensis* (MHOM/BR/LTB0016 strain) were grown at 26°C in Schneider's *Drosophila* medium pH 7.2 supplemented with 10% (v/v) heat-inactivated fetal calf serum. The number of parasites was determined by direct counting with a Neubauer chamber.

### 3. Cell proliferation

Promastigotes of *L. amazonensis* were harvested, washed twice and seed into fresh medium in the absence or in the presence of different concentration of quercetin (3 µM–96 µM) for 24 to 96 at 26°C. The cell density was estimated in a Neubauer chamber and the growth curve was initiated with 1.0×10^6^ cells/ml. The cell proliferation was verified by the counting of the cell number in a Neubauer chamber.

### 4. Determination of mitochondrial membrane potential (ΔΨ_m_)

#### 4.1. Flow cytometry studies

Promastigotes of *L. amazonensis* (1×10^6^ cells/ml) were treated for 48 hours with or without 24 µM or 96 µM quercetin and then incubated with 10 µg/ml rhodamine 123 for 20 minutes. Samples were kept on ice until analysis. Data acquisition and analysis were performed using a FACSCalibur flow cytometer (Becton Dickinson, Franklin Lakes, USA) equipped with the Cell Quest software (Joseph Trotter, Scripps Research Institute, La Jolla, USA). A total of 10,000 events were acquired in the region previously established as corresponding to the parasites. Alterations in the fluorescence for Rh123 were quantified using an index of variation (IV) obtained by the equation (MT−MC)/MC, where MT is the median of fluorescence for treated parasites and MC is the median of control parasites. Negative IV values correspond to depolarization of the mitochondrial membrane [16], [17].

#### 4.2. JC-1

The cationic JC-1 was used as a probe to determine the mitochondrial membrane potential (ΔΨ_m_) as described [9]. Promastigotes (1×10^6^ cells/ml) were cultured for 48 hours in the absence or presence of 24 µM or 96 µM quercetin. Cells were harvested, re-suspended in Hank's Balanced Salt Solution (HBSS) and the cell number was counted in a Neubauer chamber. Promastigotes (2×10^6^ cells/ml) were incubated with JC-1 (10 µg/ml) for 10 minutes at 37°C. After washing twice with HBSS, fluorescence was measured spectrofluorometrically at both 530 nm and 590 nm using an excitation wavelength of 480 nm. The ratio of values obtained at 590 nm and 530 nm was plotted as the relative ΔΨ_m_.

### 5. Alamar Blue assay

Promastigotes of *L. amazonensis* (1×10^6^ cells/ml) were treated for 48 hours with or without different concentration of quercetin (3 µM–96 µM). Cells were harvested, re-suspended in Hank's Balanced Salt Solution (HBSS) and the cell number was counted in a Neubauer chamber. Promastigotes (5×10^6^ cells/ml) were incubated with Alamar Blue (10% v/v) for 6 hours at 26°C. The absorbance was measured at 570 nm with a spectrophotometer. *L. amazonensis* cells lysed by addition of 0.1% Triton X-100 were used as positive control.

### 6. Measurement of reactive oxygen species (ROS) levels

Intracellular ROS levels were measured in treated and untreated cells. Promastigotes (1×10^6^ cells/ml) were cultured for 48 hours in the absence or presence of 24 µM or 96 µM quercetin. Promastigotes were then harvested, re-suspended in Phosphate Buffered Saline (PBS) and the cell number was counted in a Neubauer chamber. Promastigotes (2×10^6^ cells/ml) were incubated with H_2_DCFDA (20 µM) for 20 minutes at 37°C. Fluorescence was measured spectrofluorometrically at 530 nm using an excitation wavelength of 507 nm. For all measurements, basal fluorescence was subtracted. Positive control was obtained by addition of 20 units/ml glucose oxidase+60 mM glucose for 20 minutes.

### 7. Statistical analysis

All experiments were performed in triplicate. The data were analyzed statistically using Student's *t*-test and two-way analysis of variance (ANOVA). In all cases, the level of significance was set at *p*<0.05 or *p*<0.01 as indicated. The data are expressed as means ± standard errors.

## Results and Discussion

**Figure 1:**
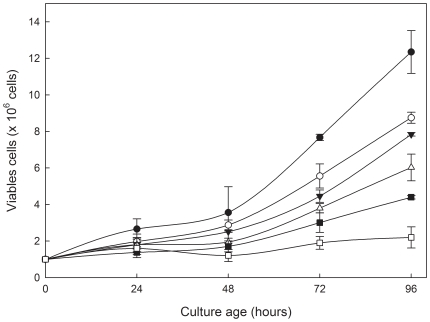
Growth of *L. amazonensis* cultivated in Schneider's *Drosophila* medium at 26°C in the absence or in the presence of Quercetin. *L. amazonensis* was cultivated in Schneider's Drosophila medium at 26°C as described in Materials and Methods for 96 h, in the absence (•) or presence of 3 µM (○), 12 µM (▾), 24 µM (Δ) 72 µM (▪) or 96 µM (□) quercetin. The number of parasites was determined by direct counting with a Neubauer chamber. Values shown are the mean±standard error of three different experiments. In the control curve (absence of quercetin), the same volume of vehicle (DMSO 0.2%) was added to the growth medium. No differences were observed on lengths of flagella and cell body of promastigote in the presence of quercetin when compared with parasite growth in the absence of quercetin.

Quercetin, an abundant naturally occurring flavonoid, has generated considerable interest as a pharmaceutical compound with a wide range of therapeutic activities. One such activity is exhibited against the bloodstream parasite *Trypanosoma brucei* and amastigotes of *Leishmania donovani* [11], [18]. To determine the effect of quercetin on the growth of *L. amazonensis*, we incubated the parasites with different concentrations of quercetin (3 µM–96 µM) for 24 to 96 hours. Incubation with quercetin inhibited *L. amazonensis* promastigote growth in a time- and dose-dependent manner (Fig. 1). This inhibitory effect was equal to 66% after 48 hours with 96 µM. The IC_50_ for quercetin at 48 hours was 31.4 µM. Total growth arrest was observed after incubating *L. amazonensis* promastigotes with 96 µM quercetin at 96 hours. This result demonstrates the antileishmanial activity of quercetin against *L. amazonensis*.

Mitochondria are essential cellular organelles that play a central role in energy metabolism and are critical for the survival of any cell. During oxidative phosphorylation, electrons are moved thorough the mitochondrial respiratory chain, and a proton gradient is established across the inner mitochondrial membrane as the energy source for ATP production. The maintenance of mitochondrial membrane potential (ΔΨ_m_) is vital for this metabolic process as well as for cell survival [3], [19]. Studies have shown that variations in ΔΨ_m_ induced by drugs are associated with cell survival in *Trypanosoma cruzi* [9], [16], *Leishmania donovani* [19] and *L. amazonensis* [20].

**Figure 2:**
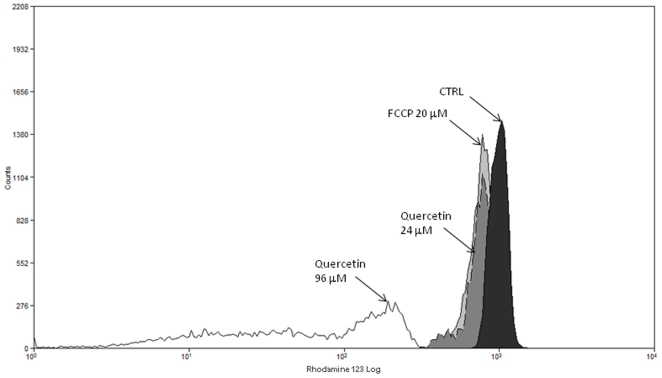
Flow cytometry analysis of *Leishmania amazonensis* treated with quercetin. *Leishmania amazonensis* was cultivated in Schneider's *Drosophila* medium at 26°C as for 48 h, in the absence or in the presence of 24 µM and 96 µM quercetin. Promastigotes were subsequently labeled with rhodamine 123 (10 µg/ml). Data acquisition and analysis were performed using a FACSCalibur flow cytometer (equipped with the Cell Quest software). A total of 10,000 events were acquired in the regions previously established as those corresponding to each form of *L. amazonensis*. Positive control was obtained by addition of FCCP (20 µM) for 20 minutes. In the control (absence of quercetin) the same volume of vehicle (DMSO 0.2%) was added to the growth medium.

To determine if quercetin induces an alteration in the ΔΨ_m_ of *L. amazonensis* promastigotes, we measured the ΔΨ_m_ by using the fluorescent probe rhodamine 123, which accumulates within energized mitochondria, in conjunction with flow cytometry analysis. Figure 2 shows that *L. amazonensis* treated with 24 µM or 96 µM quercetin for 48 hours decreased rhodamine 123 fluorescence and thus altered ΔΨ_m_. The quantification of this decrease was evaluated by unit IV. *L. amazonensis* treated with 24 µM or 96 µM led to IVs of −0.20±0.018 and −0.95±0.014 respectively. Similarly, a decrease in rhodamine 123 fluorescence and IV values (−0.29±0.026) was also observed following treatment with a mitochondrial uncoupling agent, carbonyl cyanide *p*-trifluoromethoxyphenylhydrazone (FCCP) at 20 µM [21], [22].

**Figure 3:**
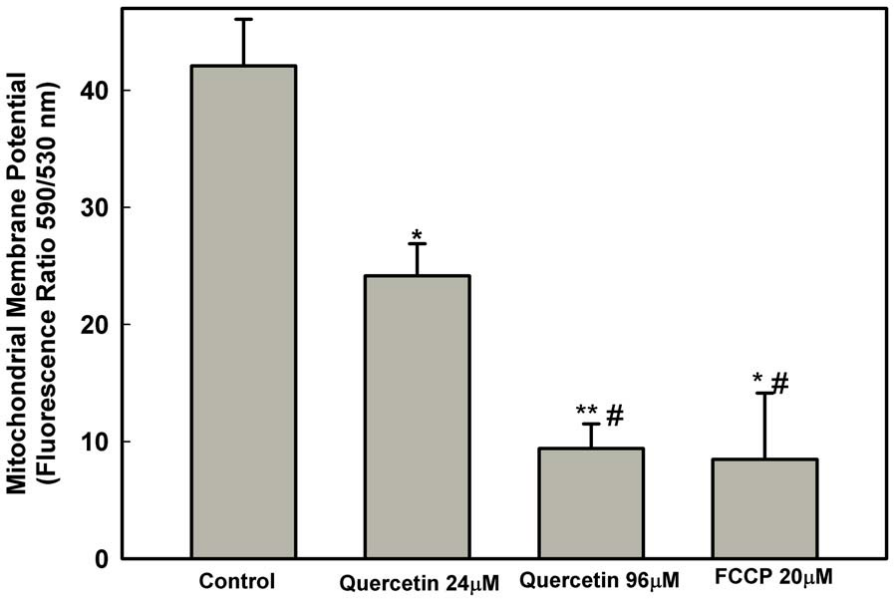
Effect of treatment of quercetin on the mitochondrial membrane potential of *Leishmania amazonensis* promastigote. *Leishmania amazonensis* was cultivated in Schneider's *Drosophila* medium at 26°C as for 48 h, in the absence or presence of 24 µM or 96 µM quercetin. Promastigotes were labeled with potentiometric probe JC-1 (10 µg/ml). Positive control was obtained by addition of FCCP (20 µM) for 20 minutes. In the control (absence of quercetin), the same volume of vehicle (DMSO 0.2%) was added to the growth medium. Dose-dependent changes in relative ΔΨ_m_ values are expressed as the ratio of the fluorescence measurement carried out at 590 nm (for J-aggregate) versus 530 nm (for J-monomer). Data are expressed as means±standard errors of three different experiments. * indicates significant difference relative to the control group (*p*<0.05); ** indicates significant difference relative to the control group (*p*<0.01); # indicates significant difference relative to the 24 µM quercetin treated group (*p*<0.05).

To confirm that quercetin induces ΔΨ_m_ collapse, we used JC-1, which is a cationic mitochondrial vital dye. This dye is lipophilic and becomes concentrated in the mitochondria in proportion to the membrane potential such that more dye accumulates in mitochondria with greater ΔΨ_m_. Spectrofluorometric data presented in Fig. 3 showed a marked decrease in relative fluorescence intensity (ΔΨ_m_ values) in a dose-dependent manner, indicating depolarization of the membrane potential in cells following treatment with 24 µM or 96 µM of quercetin, with ΔΨ_m_ reductions of 42.61% and 77.62%, respectively. Furthermore, cells incubated with 20 µM FCCP had a similar decrease in the relative fluorescence intensity values (79.82% of reduction). Taken together, these data suggest that quercetin inhibit *L. amazonensis* growth by affecting parasite mitochondrial function.

**Figure 4:**
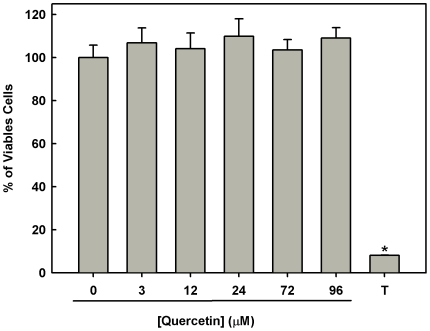
Comparison of the *L. amazonensis* untreated and treated with different concentration of quercetin. *Leishmania amazonensis* was cultivated in Schneider's *Drosophila* medium at 26°C as for 48 h in the absence or presence of different concentration of quercetin (3 µM–96 µM). Cellular viability was measured using Alamar Blue assay as described under Material and Methods. Values shown are the mean±standard error of three different experiments. In the control (absence of quercetin), the same volume of vehicle (DMSO 0.2%) was added to the growth medium. Positive control (disrupted cells) was obtained by addition of 0.1% of Triton X-100 * indicates significant difference relative to the control group (*p*<0.05). T−0.1% Triton X-100.

The reduced fluorescence of rhodamine 123 and JC-1 was not due to measuring membrane potential in the remaining live cells, because the percentage of viable cells was similar in treated and untreated parasites (Fig. 4).

**Figure 5:**
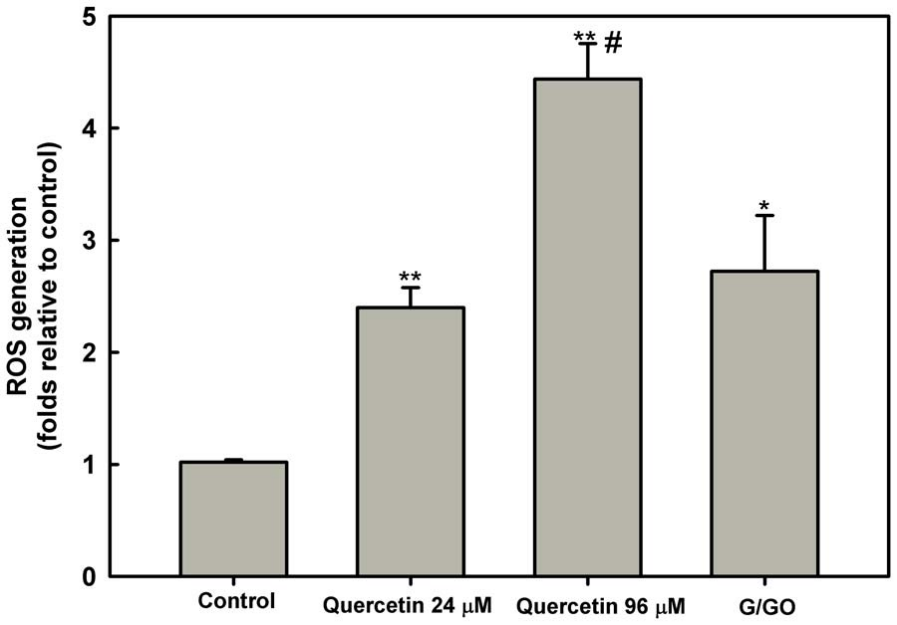
Quercetin induced ROS formation. *Leishmania amazonensis* was cultivated in Schneider's *Drosophila* medium at 26°C as for 48 h in the absence or presence of 24 µM or 96 µM quercetin. Generation of ROS was measured using fluorescent dye H_2_DCFDA as described under Materials and Methods. Data are expressed as fold increase in ROS production relative to control. Values shown are the mean±standard error of three different experiments. Positive control was obtained by addition of 20 units/ml glucose oxidase+60 mM glucose for 20 minutes. * indicates significant difference relative to the control group (*p*<0.05); ** indicates significant difference relative to the control group (*p*<0.01); # indicates significant difference relative to the 24 µM quercetin treated group (*p*<0.05). G/GO−Glucose+Glucose oxidase.

In mammalian cells, increased cellular ROS production has been suggested to be responsible for the depolarization of ΔΨ_m_ and subsequent cell death [23], [24]. To investigate if the mitochondrial dysfunction observed in *L. amazonensis* promastigote treated with quercetin is promoted by ROS production, we measured ROS levels using the cell-permeable dye H_2_DCFDA [25]–[27]. The levels of ROS in quercetin-treated cells were 2.4-fold and 4.4-fold higher for 24 µM and 96 µM, respectively, compared to the level of ROS in control cells throughout the experiment (Fig. 5). Since glucose oxidase catalyses the oxidation of D-glucose generating H_2_O_2_, it was employed as a positive control. Glucose/glucose oxidase led to an increase in ROS levels compared to control (2.6-folds, compared to the level of ROS in the control).

**Figure 6:**
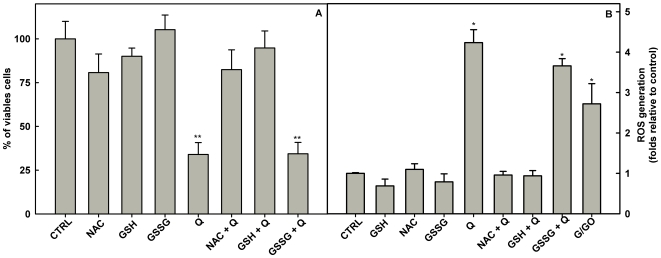
Effect of N-Acetyl-L-cysteine, Reduced Glutathione and Oxidized Glutathione on quercetin-induced cell death (A) and ROS formation (B). *L. amazonensis* was cultivated in Schneider's *Drosophila* medium at 26°C as described in Materials and Methods for 48 h in the presence of N-Acetyl-L-cysteine, reduced glutathione and oxidized glutathione in the absence or in the presence of quercetin. N-Acetyl-L-cysteine, reduced glutathione and oxidized glutathione were solubilized in PBS and quercetin was solubilized in DMSO. N-Acetyl-L-cysteine, reduced glutathione and oxidized glutathione were added to the culture to a final concentration of 300 µM, and quercetin was added to the culture to a final concentration of 96 µM. Values shown are the mean±standard error of three different experiments. In the control (absence of quercetin), the same volume of vehicle (DMSO 0.2%) was added to the growth medium. Generation of ROS was measured using fluorescent dye H_2_DCFDA as described under Materials and Methods. Data are expressed as fold increase in ROS production relative to control. Values shown are the mean±standard error of three different experiments. Positive control for ROS generation was obtained by addition of 20 units/ml glucose oxidase+60 mM glucose for 20 minutes. CTRL - control; NAC - N-Acetyl-L-cysteine; GSH - Reduced Glutathione; GSSG - Oxidized Glutathione, Q−Quercetin and G/GO−Glucose+Glucose oxidase. ** indicates significant difference relative to the control group (*p*<0.01); * indicates significant difference relative to the control group (*p*<0.05).

Reduced glutathione (GSH) is an important molecule for protecting kinetoplastids from ROS or toxic compounds, and intracellular GSH levels can be increased by the antioxidant compound N-Acetyl-L-cysteine (NAC) [28], [29]. In *L. amazonensis*, glutathione is a component of trypanothione, a major antioxidant of this parasite [30]. Thus, we tested if pre-incubation of *L. amazonensis* promastigotes with GSH or NAC (300 µM) could prevent the inhibitory effect of quercetin and found that these intracellular antioxidants protected *L. amazonensis* from inhibition by quercetin (Fig. 6A) and reduced the levels of ROS in quercetin-treated cells (Fig. 6B). Alternatively, oxidized glutathione (GSSG) did not reduce the levels of ROS and did not protect the growth inhibition promoted by quercetin. Taken together, these results indicate that inhibition of growth and disruption of ΔΨ_m_ promoted by quercetin in *L. amazonensis* is mediated by ROS production which might alter the cellular redox status.

Mitochondria are responsible for respiration and oxidative phosphorylation in eukaryotes, including trypanosomes, and they provide ATP through respiratory-coupled oxidative phosphorylation [31], [32]. The mitochondria of *Leishmania* exhibit a unique structure and are functionally distinct from mammalian mitochondria, making this organelle an exceptional chemotherapeutical target. Here, we demonstrate for the first time that quercetin exerts its antileishmanial effect on *L. amazonensis* promastigotes by generating ROS and affecting parasite mitochondrial function.

To elucidate the mechanism of cell death induced by quercetin, we chose to measure the mitochondrial membrane potential because previous studies have shown that the single mitochondrion of the kinetoplastid parasite is a good indicator of cellular dysfunction [16], [19], [33]. *L. amazonensis* promastigotes were incubated with different concentrations of quercetin for 48 hours, the shortest time that demonstrated inhibition of cellular growth (66% of inhibition). The concentrations of quercetin used in these experiments were based on the IC_50_ for 48 hours (31.4 µM). A decrease in rhodamine 123 fluorescence intensity and the JC-1 fluorescence ratio when *L. amazonensis* was treated with different concentrations of quercetin suggests increased proton permeability across the inner mitochondrial membrane with increasing quercetin. This could decrease ATP synthesis and result in parasite death. Collapse of ΔΨ_m_ was observed in *Leishmania* spp. [20], [25]–[27] and *T. cruzi* [16], [17], [34] after treatment with different drugs.

It has been demonstrated that quercetin inhibited the growth of *Leishmania donovani* promastigotes, as well as, amastigote in infected macrophages in vitro, induced topoisomerase II mediated linearization of kDNA, causing cell cycle arrest, leading to apoptosis [35]. However, quercetin has been described as a pro-oxidant, generating ROS which are responsible for cell death in some cancer cells [12], [36]. ΔΨ_m_ loss can be brought about by ROS added directly *in vitro* or induced by chemical agents [37], [38].

In this work, quercetin increased ROS generation in a dose-dependent manner after 48 hours of treatment. The antioxidant GSH and NAC each significantly reduced quercetin-induced cell death. Therefore, we suggest that quercetin-induced cell death is attributable to ROS generation could lead to ΔΨ_m_ collapse. These results are consistent with findings in *L. donovani* that the presence of ROS caused mitochondrial depolarization [39].

In conclusion, our study demonstrates, for the first time, ROS production due to quercetin treatment of *L. amazonensis* and suggests the mechanism of action for quercetin to potently inhibit cell growth in *L. amazonensis.*
